# Longitudinal Association of Sleep Quality and Duration With Motoric Cognitive Risk Syndrome in Chinese Older Adults

**DOI:** 10.1093/geroni/igaf122.1965

**Published:** 2025-12-31

**Authors:** Yiming Qiu, Youngmin Cho, Junxin Li

**Affiliations:** Johns Hopkins University, Baltimore, Maryland, United States; Johns Hopkins University, Baltimore, Maryland, United States; Johns Hopkins University, Baltimore, Maryland, United States

## Abstract

Motoric Cognitive Risk Syndrome (MCR), defined by the co-occurrence of subjective cognitive complaints and slow gait, is an established risk factor for dementia and other adverse health outcomes in older adults. While sleep characteristics have been cross-sectionally linked to MCR, their longitudinal impact remains unclear. This study examines the association between sleep characteristics and MCR risk using data from 4,359 participants (median age: 66; 50% female) without MCR at baseline from the China Health and Retirement Longitudinal Study (CHARLS), spanning baseline (2011), Wave 2 (2013), and Wave 3 (2015). Self-reported poor sleep was categorized as rarely (≤2 days/week), occasionally (3–4 days/week), and frequently (5–7 days/week); nighttime sleep duration was classified as < 6 hours/night, 6–8 hours/night, and >8 hours/night. Using Cox proportional hazard models, we found that frequent poor sleepers had higher odds of developing MCR compared to those with rare poor sleep (HR = 1.30, 95% CI: 1.00–1.69). Short sleep duration (<6 h/night) was also associated with increased MCR risk compared to 6–8 h/night (HR = 1.33, 95% CI: 1.06–1.66). Participants experiencing both frequent poor sleep and short sleep duration had the highest risk of developing MCR (HR = 1.49, 95% CI: 1.10–2.03) compared to those who rarely experience poor sleep and with 6–8 h/night sleep. These findings highlight the importance of both self-perceived sleep quality and sleep duration in MCR risk and suggest that addressing both aspects through targeted sleep interventions may help prevent mobility and cognitive decline in aging populations.

